# Human Leucocyte Antigen Class II Risk and Protective Alleles in Women with Cervical Intraepithelial Neoplasia

**DOI:** 10.15388/Amed.2024.31.1.1

**Published:** 2024-02-27

**Authors:** Olga Plisko, Jana Žodžika, Irina Jermakova, Inta Liepniece-Karele, Jeļena Eglīte, Dace Rezeberga

**Affiliations:** 1 Department of Obstetrics and Gynaecology, Riga Stradins University, Riga, Latvia; Gynaecological Clinic, Riga East University Hospital, Riga, Latvia; 2 Gynaecological Clinic, Riga East University Hospital, Riga, Latvia; 3 Pathology Centre, Riga East University Hospital, Riga, Latvia; Department of Pathology, Riga Stradins University, Riga, Latvia; 4 Joint Laboratory of Clinical Immunology and Immunogenetics, Riga Stradins University, Riga, Latvia

**Keywords:** cervical intraepithelial neoplasia, human leucocyte antigen, HLA, high-grade cervical lesions, gimdos kaklelio intraepitelinė neoplazija, žmogaus leukocito antigenas, HLA, dideli gimdos kaklelio pažeidimai

## Abstract

**Background:**

Persistent human papillomavirus (HPV) infection is a necessary cause for development of cervical precancerous lesions and cervical cancer, however, only a small percentage of women progress to cervical cancer. The local immune response, determined, among other factors, by Human Leucocyte Antigen (HLA) genes, is thought to be significant. Still the results of genome studies are inconsistent and differ between ethnical populations. The aim of the study was to assess an association between HLA-DQA1*; DQB1*; DRB1* allele’s genetic variants between women with cervical precancerous lesions and healthy controls in Latvia.

**Materials and methods:**

From January until April 2017 we enrolled 84 consecutive patients referred for colposcopy to Riga East University Hospital (Latvia) due to abnormal cervical cytology results. 57 women who came for a regular check-up and had normal cytology smears were included in the control group. Material from the cervix was taken for subsequent HLA genotyping of 13 DRB1*, 8 DQA1*, and 12 DQB1* alleles. Colposcopy was performed on all participants. In case of visual suspicion for CIN cervical biopsy was done.

**Results:**

There were 57 “no CIN” patients, 23 histologically proven CIN 1 and 61 CIN2+ cases in the study population. CIN2+ was more often associated with DQA1*0401 (OR 6.68, 95% CI 1.47-30.29, p=0.014), DRB*15 (OR 2.99, 95% CI 1.22-7.39, p=0.017), DQB1*0401 (OR 2.91, 95%CI 1.11-7.68, p=0.03), DQA1*0103 (OR 2.72, 95% CI 1.02-7.21, p=0.045), DRB1*11 (OR 2.42, 95% CI 1.10-5.33, p=0.029) and DQB1*0301 (OR 1.94, 95% CI 1.12-3.38, p=0.018). Women with “no CIN” more often had DQB1*0501 (OR 0.17, 95% CI 0.04-0.81, p=0.026), DRB1*16 (OR 0.21, 95% CI 0.06-0.78, p=0.019), DQA1*0301 (OR 0.35, 95% CI 0.14-0.87, p=0.024) and DRB1*14 (OR 0.59, 95% CI 0.01-0.46, p=0.007).

**Conclusions:**

In the current study we have demonstrated a strong association with risk and protective HLA class II alleles that are determined by the HLA-DRB1*; DQA1*; DQB1*.

## Abstract (it)

Gimdos kaklelio ikivėžinių pažeidimų atsiradimo būtinoji priežastis yra išliekanti žmogaus papilomos viruso infekcija. Tačiau tik nedaugeliui moterų išsivysto gimdos kaklelio vėžio stadija. Nustatyta, kad dalis vietinio imuninio atsako greta kitų veiksnių yra žmogaus leukocitų antigeno (HLA) genai. Tai laikoma reikšminga imuninio atsako dalimi. Tačiau genomo tyrimų rezultatai kol kas vis dar yra nenuoseklūs ir jie skiriasi priklausomai nuo tautinės populiacijos. Šio tyrimo tikslas buvo įvertinti ryšį tarp HLA-DQA1*; DQB1*; DRB1* alelių genetinių variantų moterims, kurioms buvo nustatytas gimdos kaklelio ikivėžinis pažeidimas. Latvijoje atlikto tyrimo duomenys buvo palyginti su sveikų kontrolinės grupės moterų duomenimis.

**Medžiaga ir metodai:**

į nuo 2017 m. sausio iki balandžio Rygos rytinėje universitetinėje ligoninėje (Latvija) atliekamą tyrimą buvo įtrauktos 84 moterys, kurioms atlikus kolposkopijos tyrimą gauti neįprasti gimdos kaklelio citologijos rezultatai. 57 moterys, kurios ligoninėje apsilankė profilaktiniam tyrimui ir kurių citologiniai tepinėliai neatskleidė nenormalių rezultatų, įtrauktos į kontrolinę grupę. Gimdos kaklelio medžiaga buvo paimta vėlesniam HLA genotipui nustatyti 13 DRB1*, 8 DQA1* ir 12 DQB1* alelių. Visoms tyrimo dalyvėms buvo atlikta koloskopija. Jei atliekant regimąjį tyrimą kildavo įtarimas dėl CIN (gimdos kaklelio intraepitelinės neoplazijos), buvo atliekama gimdos kaklelio biopsija.

**Rezultatai:**

Tyrimo populiacijoje buvo 57 „CIN nenustatyta“ tiriamosios bei 23 histologiškai įrodyti CIN 1 ir 61 CIN2+ atvejai. CIN2+ dažniau buvo siejama su DQA1*0401 (OR 6,68; 95 % CI 1,47–30,29; p = 0,014), DRB*15 (OR 2,99; 95 % CI 1,22–7,39; p = 0,017), DQB1*0401 (OR 2,91; 95 % CI 1,11–7,68; p = 0,03), DQA1*0103 (OR 2,72; 95 % CI 1,02–7,21; p = 0,045), DRB1*11 (OR 2,42; 95 % CI 1,10–5,33; p = 0,029) ir DQB1*0301 (OR 1,94; 95 % CI 1,12–3,38; p = 0,018). Moterims iš „CIN nenustatyta“ grupės dažniausiai buvo aptinkama DQB1*0501 (OR 0,17; 95 % CI 0,04–0,81; p = 0,026), DRB1*16 (OR 0,21; 95 CI 0,06–0,78; p = 0,019), DQA1*0301 (OR 0,35; 95 % CI 0,14–0,87; p = 0,024) ir DRB1*14 (OR 0,59; 95 % CI 0,01–0,46; p = 0,007).

**Išvados:**

Šiuo tyrimu atskleidėme stiprią sąsają tarp rizikos ir protekcinių HLA klasės II alelių, kuri nustatoma su HLA-DRB1*; DQA1*; DQB1*.

## Introduction

Cervical cancer is still a global healthcare problem despite screening and vaccination programs. It remains the fourth most common cancer in females of all ages [1]. It is known that human papillomavirus is a prerequisite, but not sufficient for the development of cervical cancer [2]. HPV is the most common sexually transmitted infection, but only a few women develop the malignant disease. The vast majority of HPV-infected women have spontaneous clearance where immune response plays a significant role [3]. Presentation of viral peptides to the host immune system is essential for an effective immune response [4]. Antigen-presenting molecules are encoded in the Human Leukocyte Antigen (HLA) class I and II genes located on the 6th chromosome [5]. Studies have assessed genetic susceptibility to cervical cancer, and several risk and protective alleles were identified. HLA DRB1*15 and DQB1*0602 alleles have been previously associated with an increased risk of cervical cancer [6–9]. Likewise, DRB1*0401, DRB1*0403, DQB1*0302, DQB1*0402, DQB1*0603 have been also described as risk alleles [10–12]. At the same time, DRB1*1301 and DQB1*0603 are commonly recognized as protective alleles [6,7,13,14]. But also, DQB1*0501 and DQA1*0301 have shown a protective effect [6,7,10,12]. The possible explanation for these variances between studies could be the gene polymorphism determined by ethnic and geographical differences [15]. Therefore, studying HLA genes related to cervical intraepithelial lesions and cervical cancer in different geographical areas is important.

This study aimed to assess an association of HLA-DQA1*; DQB1*; DRB1* allele’s genetic variants between women with cervical precancerous lesions and healthy controls in the Baltic state – Latvia.

## Materials and Methods

In the time period from January until April 2017, 84 consecutive patients referred for colposcopy to Riga East University Hospital (Latvia) due to abnormal cytology results were included in the study group. The control group consisted of consecutive 57 women who came for a check-up gynecological examination during that period of time with normal cytology results. Participants under the age of 18, pregnant women, and those who refused to participate were not included. An additional exclusion criterion was the personal history of cervical precancerous disease or cervical cancer. The study was conducted in accordance with the Declaration of Helsinki and approved by the Latvian Central Medicine Ethical Committee (Ethical approval number 3159). All patients signed informed consent before inclusion. All confidentiality and data protection principles were observed.

After the insertion of an unmoistened speculum, material from the cervix was taken using a cotton swab and then transferred to EDTA anticoagulant medium. Samples were frozen at -80 C and stored until analyzed. Colposcopy was performed by certified specialists according to the European guidelines for quality assurance in cervical cancer screening on all participants [16]. In case of visual suspicion for cervical intraepithelial neoplasia (CIN), a biopsy was taken for histological analysis in Riga East University Pathology Center. The results were classified as negative, CIN1, CIN2, CIN3 and carcinoma. All results that were equal or more severe than CIN2 were combined and analyzed together as CIN2+. In control group patients, if colposcopy was adequate and there was no sign of neoplasia it was interpreted as “no CIN”.

Subsequent analysis of frozen samples consisted of human DNA extraction, amplification, and typing of HLA genes. Extraction of human DNA was done by *QIAamp® DNA Kit (Qiagen)*. HLA genotyping of 13 DRB1*, 8 DQA1*, and 12 DQB1* alleles were performed using a programmed thermocycler (*DTLite, DNA-Technology*). The second polymorphic exone of HLA class II genes was amplified, and low-resolution real-time PCR was then used for genotyping the selected genes. In the study group 168 alleles were identified and in the control group 114 alleles.

Results were compared between “no CIN” (control), CIN1 and CIN2+ groups.

Statistical analysis of the allele frequencies, odds ratios (OR), confidence intervals (CI), and p-values was calculated with IBM SPSS 20.0 using Chi-square or Fisher’s exact test. A p-value <0.05 was considered statistically significant.

## Results

The mean age of the participants in the study group was 36.3 (±9.4) years and 35.4 (±9.3) in the control group. There were 57 “no CIN” patients, 23 histologically proven CIN 1 and 61 CIN2+ cases in the study population. We have analyzed 114 alleles in “no CIN” women, 46 in CIN1 and 122 in CIN2+. There were no differences between the groups in terms of age, education level, relationship status, and number of deliveries (Table 1).

**Table 1:** Characteristics of the groups

Parameter	No CIN, n (%)	CIN 1, n (%)	CIN 2+, n (%)	p-value
Mean age	36.3±9.4	36.4±9.7	35.1±9.2	ns
Education				
Primary	1 (1.8)	0 (0)	1 (1.6)	ns
Secondary	18 (31.6)	11 (47.8)	28 (45.9)	
Higher	38 (66.7)	12 (52.2)	32 (52.5)	
Relationship				
Registered marriage	31 (54.4)	14 (60.9)	32 (52.5)	ns
Not registered marriage	20 (35.1)	6 (26.1)	23 (37.7)	
lonely	6 (10.5)	3 (13.0)	6 (9.8)	
Number of deliveries				
No deliveries	22 (38.6)	9 (39.1)	17 (27.9)	ns
1	19 (33.3)	9 (39.1)	21 (34.4)	
2	11 (19.3)	2 (8.7)	19 (31.1)	
≥3	5 (8.8)	3 (13.0)	4 (6.6)	

The most frequently found alleles in the “no CIN” and CIN1 groups were DQB1*0602-8 (30/114, 26.3% and 18/46, 39.1%, respectively) and DQB1*0301 (30/114, 26.3% and 13/46, 28.3% respectively), but in CIN2+ group DQB1*0301 (50/122, 41,0%) and DQA1*0501 (33/122, 27.0%). (Table 2).

**Table 2:** HLA-DRB1*/DQA1*/DQB1* distribution.

Alleles	No CIN, n (%)	CIN 1, n (%)	CIN 2+, n (%)	p-value No CIN vs. CIN 1	p-value No CIN vs. CIN2+	p-value CIN1 vs. CIN2+
DRB1*01	16 (14)	9 (19.6)	21 (17.2)	0.38	0.50	0.72
DRB1*04	17 (14.9)	4 (8.7)	27 (22.1)	0.29	0.16	0.045
DRB1*07	6 (5.3)	3 (6.5)	9 (7.4)	0.71	0.51	1.00
DRB1*08	5 (4.4)	0	2 (1.6)	0.32	0.27	1.00
DRB1*09	2 (1.8)	0	0	1.00	0.23	
DRB1*10	2 (1.8)	0	0	1.00	0.23	
DRB1*11	10 (8.8)	7 (15.2)	23 (18.9)	0.26	0.03	0.58
DRB1*12	5 (4.4)	1 (2.2)	1 (0.8)	0.67	0.11	0.47
DRB1*13	7 (6.1)	1 (2.2)	2 (1.6)	0.44	0.09	1.00
DRB1*14	14 (12.3)	0	1 (0.8)	0.01	<0.0001	1.00
DRB1*15	7 (6.1)	12 (26.1)	20 (16.4)	<0.0001	0.01	0.15
DRB1*16	12 (10.5)	0	3 (2.5)	0.02	0.01	0.56
DRB1*17	10 (8.8)	9 (19.6)	13 (10.7)	0.06	0.63	0.13
DQA1*0101	14 (12.3)	8 (17.4)	14 (11.5)	0.39	0.85	0.31
DQA1*0102	23 (20.2)	17 (37)	23 (18.9)	0.03	0.80	0.01
DQA1*0103	6 (5.3)	2 (4.3)	16 (13.1)	1.00	0.04	0.16
DQA1*0201	20 (17.5)	5 (10.9)	16 (13.1)	0.29	0.34	0.69
DQA1*0301	17 (14.9)	1 (2.2)	7 (5.7)	0.02	0.02	0.45
DQA1*0401	2 (1.8)	1 (2.2)	13 (10.7)	1.00	0.01	0.12
DQA1*0501	29 (25.4)	12 (26.1)	33 (27.0)	0.93	0.78	0.90
DQA1*0601	3 (2.6)	0	0	0.56	0.11	
DQB1*0201-2	19 (16.7)	11 (23.9)	17 (13.9)	0.29	0.56	0.12
DQB1*0301	30 (26.3)	13 (28.3)	50 (41.0)	0.80	0.02	0.13
DQB1*0302	7 (6.1)	1 (2.2)	6 (4.9)	0.44	0.68	0.68
DQB1*0303	5 (4.4)	0	2 (1.6)	0.32	0.27	1.00
DQB1*0401-2	6 (5.3)	2 (4.3)	17 (13.9)	1.00	0.03	0.08
DQB1*0501	10 (8.8)	1 (2.2)	2 (1.6)	0.18	0.01	1.00
DQB1*0502-4	4 (3.5)	0	0	0.58	0.05	
DQB1*0601	3 (2.6)	0	2 (1.6)	0.56	0.68	1.00
DQB1*0602-8	30 (26.3)	18 (39.1)	26 (21.3)	0.11	0.37	0.02

HLA – Human Leukocyte Antigen CIN – Cervical Intraepithelial Neoplasia

CIN2+ was more often associated with DQA1*0401 (OR 6.68, 95% CI 1.47–30.29, p=0.014), DRB*15 (OR 2.99, 95% CI 1.22–7.39, p=0.017), DQB1*0401 (OR 2.91, 95% CI 1.11–7.68, p=0.03), DQA1*0103 (OR 2.72, 95% CI 1.02–7.21, p=0.045), DRB1*11 (OR 2.42, 95% CI 1.10–5.33, p=0.029) and DQB1*0301 (OR 1.94, 95% CI 1.12–3.38, p=0.018). Women with “no CIN” more often had DQB1*0501 (OR 0.17, 95% CI 0.04–0.81, p=0.026), DRB1*16 (OR 0.21, 95% CI 0.06–0.78, p=0.019), DQA1*0301 (OR 0.35, 95% CI 0.14–0.87, p=0.024) and DRB1*14 (OR 0.59, 95% CI 0.01–0.46, p=0.007) (Table 3).

**Table 3:** Risk estimates of CIN2+ compared to “no CIN”.

Alleles	Odds ratio (OR)	95% Confidence Interval (CI)	p-value
DRB1*11	2.42	1.10–5.33	0.029
DRB1*14	0.59	0.01–0.46	0.007
DRB1*15	2.99	1.22–7.39	0.017
DRB1*16	0.21	0.06–0.78	0.019
DQA1*0103	2.72	1.02–7.21	0.045
DQA1*0301	0.35	0.14–0.87	0.024
DQA1*0401	6.68	1.47–30.29	0.014
DQB1*0301	1.94	1.12–3.38	0.018
DQB1*0401	2.91	1.11–7.68	0.03
DQB1*0501	0.17	0.04–0.81	0.026

CIN – Cervical Intraepithelial Neoplasia

## Discussion

This is the first study describing the association between HLA class II genotypes and different cervical intraepithelial neoplasia grades in women of the one of the Baltic states – Latvia. We found a significant association between DRB1*11, DRB1*15, DQA1*0103, DQA1*0401, DQB1*0301 and DQB1*0401 and high-grade cervical intraepithelial neoplasia (CIN2+). In turn, HLA DRB1*14, DRB1*16 and DQA1*0301 were more often observed in healthy “no CIN” women.

Previous studies have described DRB1*15 association with cervical cancer in various populations [6–9,17, 18]. We have found this allele significantly more often in CIN1 and CIN2+ patients. DQA1*0401 allele was found 6-fold more often in CIN2+ compared to the “no CIN” group, however, the 95% confidence interval is wide, probably, indicating the small sample size. This allele has not been previously linked to cervical disease. Another allele showing a significant association with CIN2+ was DQB1*0401. We were able to find that this allele was mentioned only in one study and was found in 2/47 healthy patients [8]. DRB1*11 showed a more than 2-fold increased risk of CIN 2+, a similar finding was observed in the British population [10]. DQB1*0602, earlier described as a risk allele in some genome-wide association studies, was seen almost equally often in no CIN and CIN2+ groups, but it was more frequent in CIN1 patients than CIN2+ [6,7]. DQB1*0301 previously associated with high-grade CIN and cervical cancer in British, Spanish and Norwegian populations, in our study was also seen more often in CIN2+ compared to healthy women [10–12,19].

Other European population studies have identified DRB1*0401, DRB1*0403, DQB1*0302, DQB1*0402, DQB1*0603 alleles as related to increased risk of CIN and cervical cancer, however, we were not able to repeat these findings [10–12].

We identified 3 HLA alleles DRB1*14, DRB1*16 and DQA1*0301 significantly more often in the “no CIN” group compared to both CIN1 and CIN2+. None of these has been reported earlier. Additionally, DQB1*0501 has shown a protective effect against CIN2+. Similar finding was observed in the British population [10,12]. DRB1*1301 and DQB1*0603 were associated with a protective effect in many previous studies [6,7,13,14,20, 21], although our data did not provide such association. DQA1*0103 was also previously described as protective allele, however in our data set it was 2.5-fold more often seen in CIN2+ than in “no CIN” patients [6,7].

Some common HLA alleles were found in different studies, but still, the inconsistency is observed. HLA gene polymorphism highly depends on the ethnic origin of the studied population [15]. This could explain variances in the HLA genotypes associated with the risk or protection against cervical cancer. We propose, that despite numerous previous studies, it is important conducting similar research in the populations not described before. Along with the HLA genotypes identified throughout different ethnic groups, there could be specific genes characteristic for each one.

Since HPV infection is extremely common, but only some women develop high-grade cervical intraepithelial lesions and cervical cancer, it is important to make an individual risk prediction. There have already been some models described, that included HPV type, smoking, sexual habits, demographic factors and medical history [22-24]. There has also been an attempt to implement results of HLA genotyping in such a prediction tool [25]. Results of these studies are very promising and such investigations should be continued. There will be a need to test these prediction models in different populations.

The strength of this study is that all women, including the control group, had a colposcopy, and the severity of the lesion was confirmed by histological analysis. The major limitation is the small number of participants, due to this reason we cannot make any assumption about the association between HLA haplotypes and cervical intraepithelial neoplasia.

In conclusion, we have identified some of the HLA DQA1*; DQB1*; DRB1* alleles related to increased or decreased risk of cervical intraepithelial neoplasia and cervical cancer previously described in other studies. However, some new associations were described for the first time. To prove our data and identify HLA haplotypes linked to the development of cervical cancer in Latvian population, the study should be continued with a greater number of participants, preferably also including data from other two Baltic states.
